# Hemostatic effect of oxidized regenerated cellulose vs. topical tranexamic acid in total knee arthroplasty—a prospective randomized controlled trial

**DOI:** 10.3389/fsurg.2024.1515610

**Published:** 2025-01-07

**Authors:** Bowei Li, Wenjie Pan, Jianbing Ma, Yuanchi Huang

**Affiliations:** Department of Knee Joint Surgery, Xi'an Honghui Hospital, Xi'an Jiaotong University, Xi'an, Shaanxi, China

**Keywords:** tranexamic acid, oxidized regenerated cellulose, total knee arthroplasty, total blood loss, hemoglobin drop

## Abstract

**Aims:**

This study compared the hemostatic effects and complications of oxidized regenerated cellulose (ORC) and topical TXA in total knee arthroplasty (TKA), thus providing a reference for the use of ORC as an alternative hemostatic agent to TXA in TKA.

**Methods:**

A total of 105 patients were included in this study and randomized into blank control, ORC, and TXA groups. The primary outcomes were total blood loss, hemoglobin drop (Hb drop), transfusion rates, and incidence of thrombosis. The secondary outcomes included operation time, tourniquet duration, coagulation parameters, inflammation markers, and complication rates.

**Results:**

Total blood loss was 1,002.47 ± 308.58 ml and 964.68 ± 273.00 ml in the ORC and TXA groups, respectively, both significantly lower than that in the blank control group (1,168.94 ± 405.04 ml) (*P*_1_ = 0.043 and *P*_2_ = 0.014, respectively). Hb Drop was statistically insignificantly different between the ORC (36.03 ± 12.17 g/L) and TXA (34.32 ± 10.19 g/L) groups (*P* = 0.555). There was no statistically significant difference in transfusion rate, operation time, tourniquet duration, coagulation parameters, inflammation markers, and complication rates among the three groups.

**Conclusion:**

In conclusion, our prospective randomized controlled trial (RCT) highlights that, oxidized regenerated cellulose (ORC) can reduce postoperative invisible blood loss in total knee arthroplasty and achieve a hemostatic effect similar to topical tranexamic acid (TXA). This provides a safe and effective hemostatic option for patients with severe underlying diseases or contraindications to tranexamic acid.

**Clinical Trial registration:**

https://www.chictr.org.cn/bin/project/edit?pid=186370, identifier (ChiCTR2200066633).

## Introduction

After nonoperative treatment options, patients with end-stage osteoarthritis and knee joint disease generally pursue total knee arthroplasty (TKA) as an option (1, 2). However, TKA-associated blood loss remains a major concern. Specifically, intraoperative osteotomy and medullary exposure come with massive blood loss, which may affect the postoperative functional exercise of patients (3). Residual blood may even cause bone destruction, prosthesis dislocation and other complications (4–6). In addition, blood transfusion elevates the risk of bloodborne infectious diseases and transfusion-related immunological diseases in patients (7). Therefore, there is acritical clinical need to seek a hemostatic regimen for the effective minimization of perioperative blood loss in patients undergoing TKA (8).

Tranexamic acid (TXA) is a synthetic derivative of the amino acid lysine, with broad clinical applicability for its sound hemostatic effect and economic benefits (9). In the majority of studies, TXA has been validated to be effective in hemostasis without increasing the risk of thrombotic events (10). However, TXA may not only be contraindicated in patients with TXA allergy, a history of thromboembolism, but also is associated with epilepsy, nausea, vomiting, and diarrhea (11–15). Therefore, it is essential to find a safe and effective alternative hemostatic regimen for avoiding adverse drug reactions to TXA.

Oxidized regenerated cellulose (ORC) is a type of biodegradable hemostatic material derived from plants, which is biocompatible because of carboxyl groups produced during its oxidation and, therefore, can be completely degradable and absorbable in the body (16). At present, ORC provides a physical scaffold for platelet adhesion and aggregation and also facilitates hemostasis through vasospasm and vasoconstriction caused by the local low pH without compromising the coagulation function of the body, which enables its widespread use in the fields of thoracic surgery, urology, and gynecology. Our previous study demonstrated that ORC could sustain hemostasis and effectively reduce postoperative hidden blood loss in patients receiving TKA (17).Yet, it remains undetermined about the effectiveness and safety of ORC as compared to those of topical application of TXA.

This study, therefore, analyzed the difference in the hemostatic effects of ORC and topical TXA in TKA compared to the control group. We hypothesized that ORC might have similar hemostatic effects to topical application of TXA without increasing the risk of complications.

## Methods

### Study design

This prospective randomized controlled trial (RCT) compared the effects of two topical hemostasis methods on perioperative blood loss in patients undergoing TKA, which was designed in accordance with the *Declaration of Helsinki* and the CONSORT statement. It was reviewed and approved by the Ethics Committee of the Hong hui Hospital Affiliated to Xi’an Jiao tong University (No. 202211017). Additionally, this trial was registered on the Chinese Clinical Trial Registry (ChiCTR2200066633).

### Sample size calculation

The sample size for this study was calculated based on a study by Georgiadis et al. (18),which is a prospective randomized controlled trial designed to probe the effect of topical TXA on perioperative blood loss in patients undergoing TKA. Assuming that the mean difference in perioperative blood loss between the two groups was 352 ml or greater, 26 patients were required in each group for achieving a statistical power of 0.80 and an *α* error of 0.05. Considering the exclusion and withdrawal of patients and patients with incomplete data during the trial, the study enrolled 105 patients (35 patients in each group).

### Participants

The inclusion criteria for this study are: those who were diagnosed with knee osteoarthritis at Xi'an Hong hui Hospital from December 15, 2022 to March 15, 2023, and planned to undergo initial unilateral total knee joint surgery in our hospital. Exclusion criteria for patients were as follows: patients with genu varum, genu valgum, or flexion contracture of the knee joint of greater than 30°; patients with cerebrovascular disease; patients with previous myocardial infarction; patients with heart failure (New York Heart Association class III or IV); patients with atrial fibrillation; patients with hepatic and renal insufficiency, patients with preoperative anemia; patients with a history of arterial or venous thromboembolism; patients with contraindications to TXA; patients with coagulation disorders (preoperative international normalized ratio > 1.4, activated partial thromboplastin time > 1.4 × normal value, and platelets <140,000/mm^3^); and patients with intraoperative complications (fracture, etc.). In addition, patients who withdrew or were lost to follow-up during the trial were excluded from the study. This work was designed in accordance with the Declaration of Helsinki and reviewed and approved by the Ethics Committee of our hospital. Informed consent was obtained from all patients involved in the study. After the patient signed the informed consent, demographic data (BMI, ASA grade, platelet count) and preoperative coagulation indicators (PT, APTT, TT, FIB) of the patient were collected (Table 1).

**Table 1:** Baseline characteristics of patients in the three groups.

	Control group	ORC group	TXA group	*P* value
	*n* = 33	*n* = 34	*n* = 34	
Gender (male/female, *n*)	11/22	7/27	11/23	0.436
Age (year)	69.48 ± 6.08	66.88 ± 5.31	67.21 ± 5.72	0.133
BMI (kg/m^2^)	25.23 ± 4.07	25.41 ± 3.10	25.23 ± 2.88	0.969
ASA grade (Ⅱ/Ⅲ, *n*)	25/8	27/7	21/13	0.230
Preoperative-Hb (g/L)	139.24 ± 13.65	137.26 ± 16.59	140.85 ± 14.59	0.616
Preoperative-Hct (%)	41.63 ± 3.71	41.44 ± 4.55	42.18 ± 3.74	0.731
Platelet count (×10^9^/L)	202.91 ± 53.14	228.47 ± 62.11	233.88 ± 62.98	0.082
Preoperative coagulation index				
PT (s)	12.49 ± 0.77	12.23 ± 0.68	12.12 ± 0.57	0.081
APTT (s)	29.08 ± 2.36	29.37 ± 2.76	28.47 ± 1.55	0.255
TT (s)	16.50 ± 0.56	16.49 ± 0.79	16.68 ± 0.96	0.544
FIB (g/L)	3.16 ± 0.60	3.34 ± 0.67	3.21 ± 0.57	0.485

BMI, body mass index; ASA, american society of aneshesiologists; Hb, hemoglobin; HCT, hematocrit; PT, prothrombin time; APTT, activated partial thromboplastin time; TT, thrombin time; FIB: fibrinogen; ORC, oxidized regenerated cellulose; TXA, tranexamic acid;.

### Randomization and intervention

**Figure 1:**
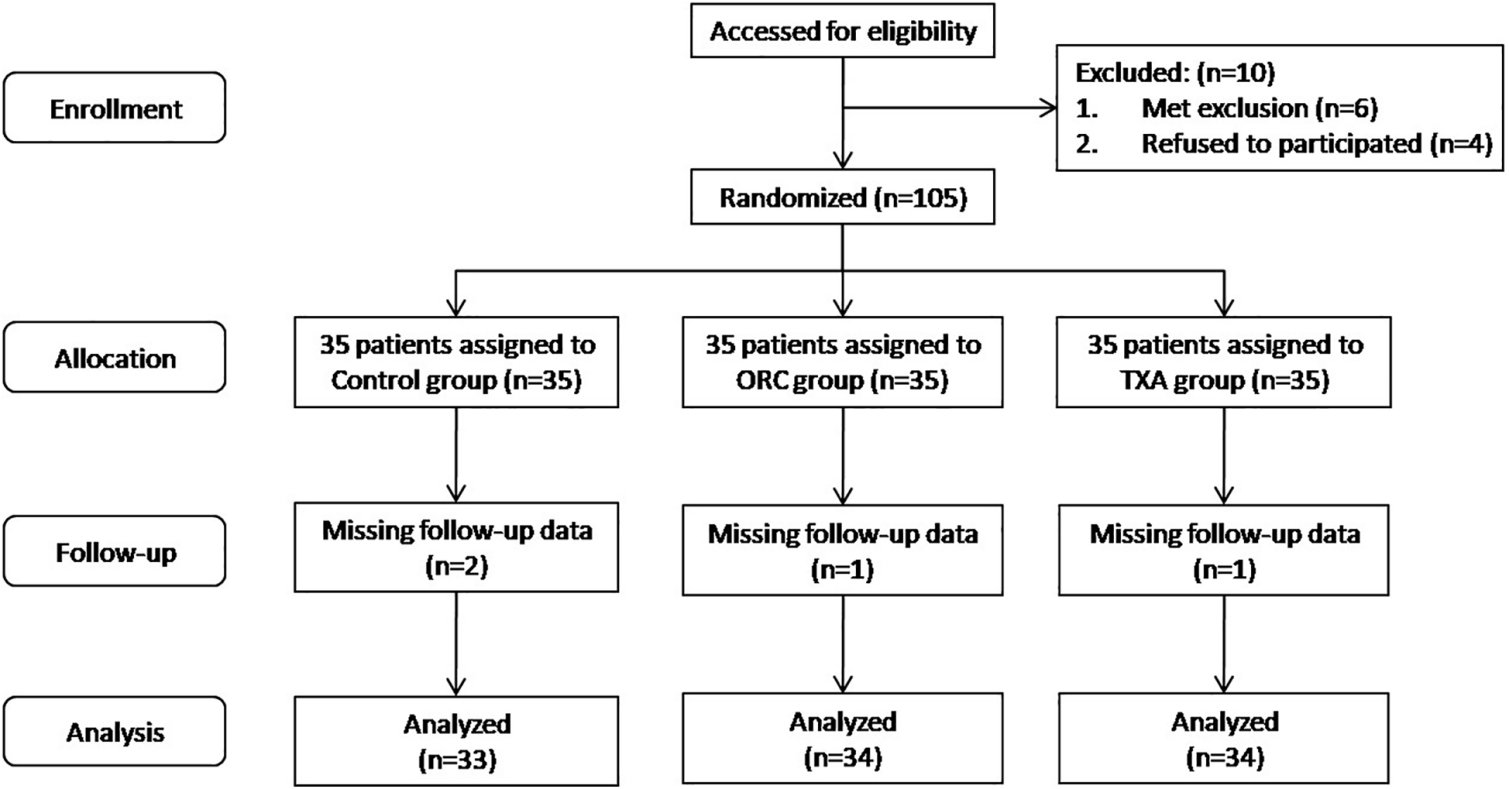
Flow chart of patient inclusion in this study.

Patients (*n* = 105) who were included in the study according to the inclusion and exclusion criteria were randomized into three groups (35 patients/group) by nurses with a random number table generated by a computer based on their admission order: a blank control group (control group),an ORC group [two pieces of ORC (SurgicelFibrillar^TM^; 2.5 cm × 5.1 cm; Ethicon Inc., San Lorenzo, Puerto Rico, USA) were placed in the knee joint], and a TXA group (patients were intra-articularly injected with 2 g of TXA).Prior to surgery, the grouping of each patient was brought into the operating room by the nurse in a sealed envelope, and then the envelope was opened by the operator to confirm the grouping of patients. The researchers analyzed the data after the completion of the trial. During the trial, 2, 1, and 1 patient withdrew from the blank control, ORC, and TXA groups, respectively. Finally, the study included a total of 101 patients (33 in the blank control group, 34 in the ORC group, and 34 in the TXA group) (Figure 1).

### Surgery procedure

**Figure 2:**
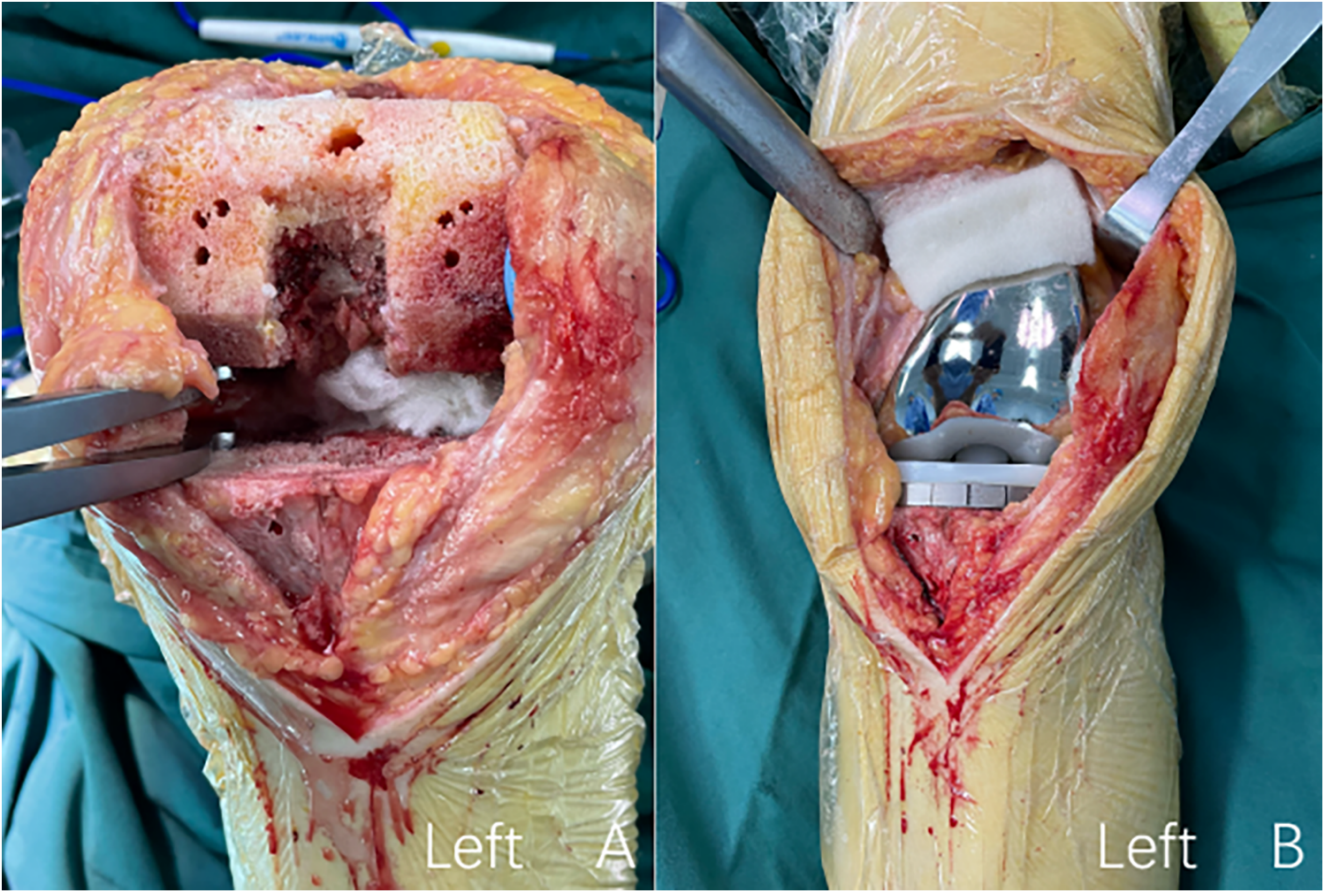
The procedure of intraoperative topical application of oxidized regenerated cellulose. **(A)** Oxidized regenerated cellulose was placed on the posterior side of the joint capsule; **(B)** oxidized regenerated cellulose was placed on the suprapatellar Bursa.

**Figure 3:**
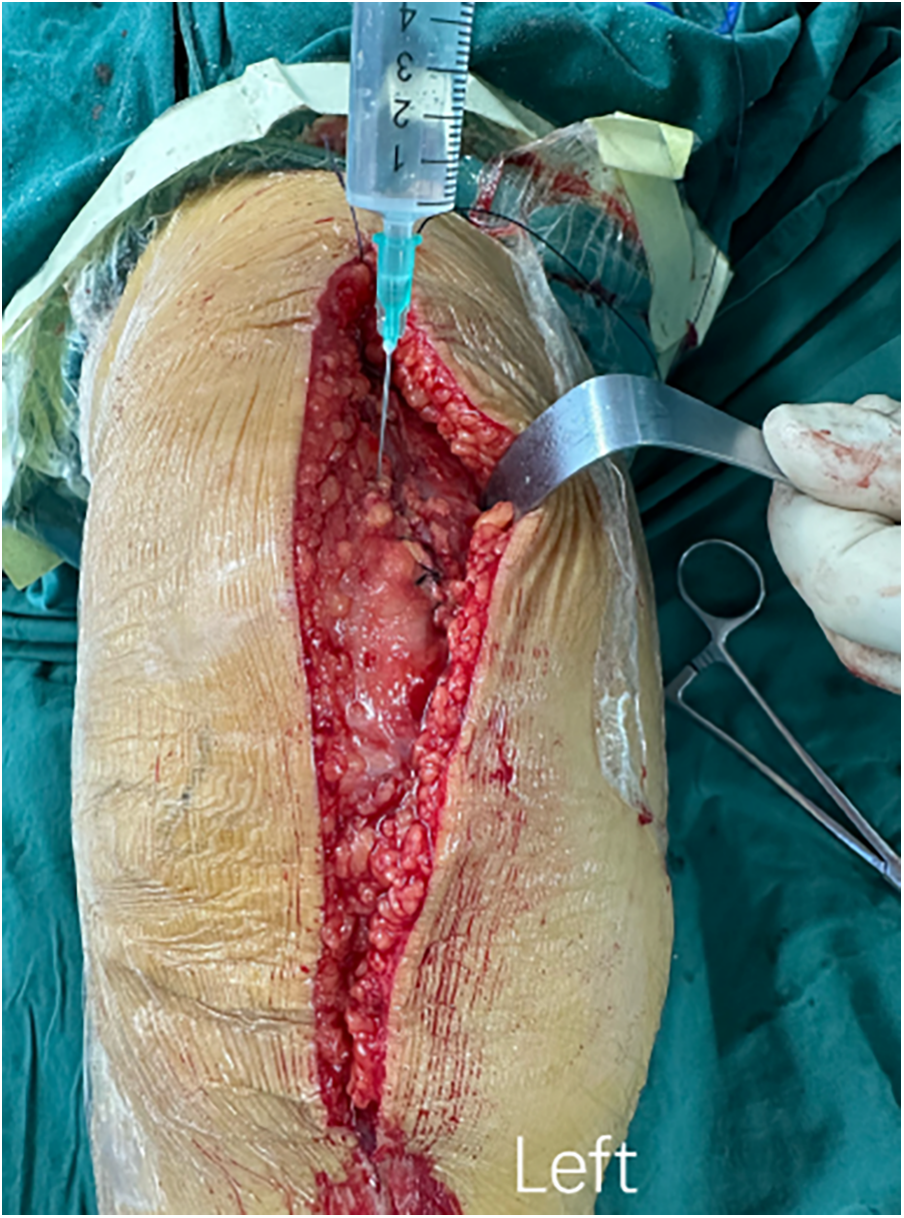
The procedure of intraoperative topical application of tranexamic acid. After the articular cavity was sutured, 20 ml of TXA at a concentration of 0.1 g/ml was diluted to 50 ml with normal saline and injected into the articular cavity.

All patients were subjected to surgery by the same attending surgeon and were treated with femoral nerve block and general anesthesia. The affected extremity was disinfected and treated with a pneumatic tourniquet. All surgeries were performed using a parapatellar approach, with an incision of about 14 cm created in the middle of the knee. After osteotomy, the cemented prosthesis (A3 GT., Beijing AK Medical Co., Ltd., Beijing, China) was implanted for all three groups. During the operation, cocktail therapy was used for local infiltration analgesia. Ropivacaine 200 mg + dexamethasone 5 mg + epinephrine 1 mg + normal saline were used for periarticular injections in the following areas: peripatellar soft tissues, quadriceps, medial and lateral collateral ligaments, and posterior joint capsule. The wound of patients in the blank control group was sutured before the pneumatic tourniquet was released. Patients in the ORC group underwent hemostasis using two pieces of ORC, with one piece on the posterior side of the articular capsule and the other piece on the suprapatellar bursa (Figure 2). The alignment of prostheses in TKA surgery is usually based on different anatomical landmarks, while our hemostatic material is placed in the joint cavity after osteotomy, so it does not affect the formulation of surgical force lines (19). Thereafter, the wound was sutured, followed by the releasing of the pneumatic tourniquet. After the articular cavity in the TXA group was sutured, 20 ml of TXA at a concentration of 0.1 g/ml was diluted to 50 ml with normal saline and injected into the articular cavity. Next, the wound was sutured, and then the pneumatic tourniquet was released (Figure 3). No drainage tubes were used for all patients. In addition, for the consideration of medical ethical issues such as patient protection, all patients in the 3 groups were given 1 g intravenous tranexamic acid 5–10 min before the tourniquet was released during the surgery.

### Postoperative care protocol

After the operation, ice packs were applied to the knee once every 4 h for 0.5 h. Encourage patients to get out of bed early and perform muscle strength training and knee mobility exercises. All patients were discharged on the third day after surgery. The same analgesic measures were used for all patients postoperatively, including a self-controlled analgesia pump (dezocine, 0.5 mg/ml, 100 ml), oral Imrecoxib Tablets (0.1 g, twice daily for 2 months), and oral Codeine (10 mg, twice daily for 2 weeks).All patients were given an intravenous drip of antibiotics (cefuroxime sodium; 1.5 g/100 ml) at 6 and 24 h postoperatively to prevent infection. The same preventive measures for vein thrombosis were used for all patients, Meanwhile, patients were subcutaneously injected with low-molecular-weight heparin calcium injection (4,100 IU in 0.4 ml, 0.4 ml/time) once daily during hospitalization until the third postoperative day and took Aspirin tablets (2.5 mg/day) once daily for 14 days after discharge. Postoperatively, the nausea and vomiting of the patients were recorded. The routine blood test of patients was conducted on postoperative days 1 and 3. Blood transfusion was given when hemoglobin (Hb) concentrations were < 70 g/L, while blood transfusion was decided according to the anemia symptom of patients when Hb concentrations were between 70 and 100 g/L. Lower-extremity venous ultrasonography was performed on postoperative day 1 and week 2 to observe the occurrence of thrombotic events in patients. The patients were reviewed and followed up on time at 2 and 6 weeks after discharge to record the occurrence of complications.

### Primary outcomes

The primary outcomes were total blood loss (TBL), hemoglobin drop (Hb drop), transfusion rates, and incidence of thrombosis. TBL was calculated using the Gross method (20): TBL = total preoperative blood volume (PBV) × [preoperative hematocrit (HCT)−postoperative HCT]/preoperative HCT. The total PBV was calculated with reference to the method of Nadler et al. (21): total PBV = k1 × height3 (m) + k2 × body mass (kg) + k3 (for men: k1 = 0.3669, k2 = 0.03219, and k3 = 0.6041; for women: k1 = 0.3561, k2 = 0.03308, and k3 = 0.1833). Hb drop was calculated with the following formula: Hb drop = preoperative Hb value−minimum Hb value during hospitalization.

### Secondary outcomes

The secondary outcomes included operation time, tourniquet duration, coagulation parameters [D-dimer and fibrin degradation products (FDPs)] and inflammation markers [C-reactive protein [CRP] and erythrocyte sedimentation rate [ESR]] on postoperative days 1 and 3, and complications (swelling, nausea and vomiting, wound complications, infections, pulmonary embolism, myocardial infarction, and readmission) within 6 weeks.

### Statistical analysis

All data in this study were analyzed using SPSS25.0 statistical software. Differences were statistically significant at *P* < 0.05.

Data with normal distribution were expressed as mean ± standard deviation, while data with skewed distribution were presented as median (interquartile range).

Categorical variables such as sex, American Society of Anesthesiologists classification, and number of patients with complications were analyzed with Pearson's chi-squared test or Fisher's exact test.

Baseline data of patients and continuous variables including TBL, Hb drop, operation time, tourniquet duration, postoperative inflammation, and coagulation parameters were compared with analysis of variance or nonparametric tests.

## Results

Demographic data and preoperative laboratory values of patients were collected and analyzed. The results showed that the three groups were comparable (Table 1).

TBL was 964.68 ± 273.00 ml in the TXA group and 1,002.47 ± 308.58 ml in the ORC group, with no statistically significant difference between the two groups (*P* = 0.641) (Table 2). Moreover, TBL in these two groups was markedly lower than that in the control group. Another primary indicator, Hb drop of patients was 34.32 ± 10.19 g/L in the TXA group, which was substantially lower than that in the control group (*P* = 0.019) and slightly lower than that in the ORC group with no statistically significant difference (*P* = 0.555) (Table 2). These data indicate that the local application of ORC and TXA in TKA is an effective means to reduce the total blood loss.

**Table 2:** Comparison of TBL, Hb drop, transfusion rates, tourniquet duration, and operation time among the three groups.

	Control group	ORC group	TXA Group	*P* value	*P*_1_	*P*_2_	*P*_3_
	*n* = 33	*n* = 34	*n* = 34				
TBL (ml)	1,168.94 ± 405.04	1,002.47 ± 308.58	964.68 ± 273.00	0.032	0.043	0.014	0.641
Hb drop (g/L)	41.21 ± 13.09	36.03 ± 12.17	34.32 ± 10.19	0.052	0.077	0.019	0.555
Blood transfusion (n)	2/33	0/34	0/34	0.122			
Tourniquet duration (min)	75.76 ± 14.42	69.71 ± 15.07	72.50 ± 14.42	0.244	0.094	0.365	0.433
Operation time (min)	97.03 ± 14.67	93.88 ± 14.21	96.56 ± 15.84	0.647	0.390	0.897	0.461

TBL, total blood loss; ORC, oxidized regenerated cellulose; TXA, tranexamic acid; *P*_1_,the control group vs. the ORC group;*P*_2_,the control group *vs.* the TXA group;*P*_3_, the ORC group vs. the TXA group.

After postoperative evaluation, postoperative blood transfusion was performed for 2 patients in the control group. No statistical difference was found in the transfusion rate among the three groups (*P* = 0.122) (Table 2).

No statistically significant difference was observed among the three groups in terms of tourniquet duration and operation time (Table 2). Consistent with previous surgical experience, shorter operation time, tourniquet duration and effective hemostasis strategies generally mean less perioperative blood loss, resulting in better surgical outcomes (22, 23).

The three groups exhibited no statistically significant differences in coagulation parameters (D-dimer and FDPs) (Table 3) and inflammation markers (CRP and ESR) (Table 4) in the preoperative period and on postoperative days1 and 3.

**Table 3:** Comparison of coagulation parameters among the three groups.

		Control group *n* = 33 M (IQR)	ORC group *n* = 34 M (IQR)	TXA group *n* = 34 M (IQR)	*P* value	*P*_1_	*P*_2_	*P*_3_
D-dimer (mg/L)	PRE-OP	0.40 (0.20)	0.39 (0.25)	0.36 (0.19)	0.626	1.000	1.000	1.000
	POD 1	3.63 (3.34)	3.15 (3.96)	3.32 (4.10)	0.838	1.000	1.000	1.000
	POD 3	1.32 (1.25)	1.34 (1.02)	1.33 (0.84)	0.847	1.000	1.000	1.000
FDPs (mg/L)	PRE-OP	1.60 (0.93)	1.72 (0.92)	1.60 (0.80)	0.591	1.000	1.000	1.000
	POD 1	11.50 (14.33)	10.30 (12.97)	10.11 (14.35)	0.735	1.000	1.000	1.000
	POD 3	4.41 (3.65)	4.67 (3.02)	4.40 (2.27)	0.806	1.000	1.000	1.000

FDP, fibrin degradation products; ORC, oxidized regenerated cellulose; TXA, tranexamic acid; PRE-OP, preoperative; POD 1, postoperative 1 day; POD 3, postoperative 3 day; *P*_1_, the control group vs. the ORC group; *P*_2_, the control group *vs.* the TXA group; *P*_3_, the ORC group vs. the TXA group.

**Table 4:** Comparison of inflammatory markers among the three groups.

		Control group *n* = 33 M (IQR)	ORC group *n* = 34 M (IQR)	TXA group *n* = 34 M (IQR)	*P* value	*P*_1_	*P*_2_	*P*_3_
CRP (mg/L)	PRE-OP	1.36 (2.33)	1.04 (2.40)	1.08 (1.52)	0.410	1.000	0.546	1.000
	POD 1	13.76 (14.94)	21.30 (23.96)	15.33 (13.97)	0.390	0.528	1.000	1.000
	POD 3	39.89 (47.94)	42.16 (49.96)	31.82 (47.82)	0.796	1.000	1.000	1.000
ESR (mm/H)	PRE-OP	10.00 (11.00)	9.50 (9.25)	11.50 (10.25)	0.566	0.928	1.000	1.000
	POD 1	14.00 (17.00)	12.50 (14.00)	14.00 (14.25)	0.275	0.347	0.836	1.000
	POD 3	23.00 (18.50)	21.00 (17.50)	24.50 (17.25)	0.464	1.000	1.000	0.647

CRP, c-reactive protein; ESR, erythrocyte sedimentation rate; ORC, oxidized regenerated cellulose; TXA, tranexamic acid; PRE-OP, preoperative; POD 1, postoperative 1 day; POD 3, postoperative 3 day; *P*_1_, the control group vs. the ORC group; *P*_2_, the control group *vs.* the TXA group; *P*_3_, the ORC group vs. the TXA group.

After a 6-week follow-up, complications were analyzed in the three groups (Table 5). The incidence of muscular vein thrombosis, swelling, wound complications, and nausea and vomiting were not statistically different among the three groups. Additionally, none of the patients experienced symptomatic lower-extremity deep vein thrombosis or pulmonary embolism. There were also no early infections or unplanned readmissions in the three groups.

**Table 5:** Complications during the 6-week follow-up period.

	Control group	ORC group	TXA group	*P* value
	*n* = 33	*n* = 34	*n* = 34	
Deep vein thrombosis	0/33	0/34	0/34	
Calf muscular vein thrombosis	15/33	14/34	17/34	0.766
Swelling	5/33	3/34	8/34	0.250
Wound complications	4/33	3/34	3/34	0.873
Nausea and vomiting	5/33	6/34	9/34	0.472
Pulmonary embolism	0/33	0/34	0/34	
Early infection	0/33	0/34	0/34	
Unplanned readmission	0/33	0/34	0/34	

ORC, oxidized regenerated cellulose; TXA, tranexamic acid.

## Discussion

In this study, it can be observed that after hemostatic measures, the total blood loss of patients within 3 days after surgery still reached nearly 1,000 ml, accounting for about 25% of the total blood volume, which is inconsistent with the observed intraoperative blood loss, a considerable part of the blood loss is hidden. TKA-associated TBL is primarily composed of two parts, namely, overt blood loss and hidden blood loss, among which overt blood loss includes intraoperative blood loss and postoperative drainage. In 1973, Pattison et al. first discovered this phenomenon during joint replacement surgery for rheumatoid arthritis patients, and was first defined in 2000 by KR Sehat et al., where the concept of invisible blood loss (HBL) was first proposed (24, 25). Since none of the patients in this study used drainage tubes, the difference in TBL among the three groups was mainly attributed to the difference in hidden blood loss. Prior studies demonstrated that high levels of free fatty acids in the postoperative blood induced oxidative stress and damaged red blood cells, contributing to a significant amount of hidden blood loss, which accounted for 73% of TBL in patients (26, 27). It is also believed that the continuous bleeding of the bone marrow cavity and tissue after the operation infiltrates the tissue space and remains in the joint cavity. It has also been suggested that perioperative anticoagulants work by interfering with the clotting mechanism, producing invisible blood loss. Therefore, it is of great clinical importance to diminish hidden blood loss as it can ameliorate postoperative swelling and pain, elevate the effectiveness of postoperative functional exercises, and improve prognosis in patients (26).

The results of this prospective RCT unveiled that compared to the blank control group, the ORC and TXA groups both showed marked reductions in TBL during TKA (*P*_1_ = 0.043, *P*_2_= 0.014). However, ORC group has no statistically significant difference with TXA group (*P*_3_ = 0.641). In addition, compared to the blank control group, the TXA group also effectively reduced the Hb drop of patients (P_2_ = 0.019), and there was no statistical difference between the TXA and ORC groups (P_3_ = 0.555). Concordant with our previous findings, that ORC can diminish ongoing postoperative hidden blood loss, then reducing TBL in TKA (17). In the present study, these results also confirm our hypothesis that in TKA, topical ORC is similarly effective in decreasing blood loss to topical TXA without causing additional risks. As an absorbable hemostatic material, ORC can boost vasoconstriction, provide a scaffold for platelet adhesion and aggregation, and form a gel substance, which jointly promotes the formation of blood clots (28, 29). In this study, with the continuous optimization of modern perioperative management strategies, the number of transfusion cases was basically effectively controlled after effective hemostatic measures.

TXA prevents activation of the tissue-type plasminogen activator (t-PA) by forming a complex with it, then inhibiting fibrinolysis and lowering blood loss (30). In our study, topical TXA exerted a good hemostatic effect (TBL, 964.68 ± 273.00 ml; *P*_2_ = 0.014) and also declined Hb Drop in patients (*P*_2_ = 0.019), consistent with most studies (9, 31).

In thoracic surgery, the use of ORC can prevent intrathoracic adhesions (32). In breast surgery, it can be used as a filler in breast-conserving surgery and used to reduce drainage volume after lymph node dissection (33, 34). Use in general surgery can reduce bleeding after hepatectomy, gastrectomy, and laparoscopic cholecystectomy (35, 36). However, implants do not have no impact on the human body. Previous literature has shown that implants can affect serum indicators, there have also been some negative reports about ORC in recent years, such as retention-related complications, which could induce radiologists to misinterpret imaging findings (37, 38). In addition, ORC also has the risk of causing allergic reactions and foreign body reactions, and the acidic environment it generates may also cause nerve fiber degeneration (39, 40). Therefore, it is important to understand the specific properties of ORC, avoid misdiagnosis, and use them sparingly.

Controversies still exist regarding the use of pneumatic tourniquets in modern knee arthroplasty and their effect on blood loss (41–43).The use of no tourniquet accelerates the recovery of patients and decreases the incidence of complications (44), whereas the use of tourniquets enhances the visibility of the operated area, maintains a clean surgical field, and shortens operation time. Shorter operation time is associated with lower incidence of infections (45, 46). In our study, tourniquets were used in all patients to avoid the influence of tourniquets on the results, and there was no statistical difference in operation time and tourniquet duration among the three groups.

Surgical trauma and tourniquet application lead to activation of the fibrinolytic system in the body, thereby producing fibrinolytic enzymes and then eliciting the release of FDPs, such as D-dimer, into the circulation (47, 48). Fibrin degradation product (FDP) is a sensitive index reflecting fibrinolysis, and its elevated level indicates that the blood is in a hypercoagulable state, reflecting the overall level of fibrinolysis (49). D-dimer is the simplest product generated after fibrin degradation, and it is the smallest fragment of the fibrin degradation product. It is considered as a molecular marker of hypercoagulability and hyper fibrin degradation *in vivo* (50). Our results displayed no statistically significant difference in levels of D-dimer and FDPs among the blank control, ORC and TXA groups from the preoperative period to postoperative day 3. This showed that the use of ORC and TXA did not exert additional effects on the fibrinolysis system in patients.

A large amount of osteotomy and soft tissue dissection during TKA can cause inflammatory response, and the inflammation caused by surgery can lead to weakened muscle strength and increased pain, further affecting postoperative functional recovery (51). Researchers have observed that besides reducing bleeding, tranexamic acid also reduces surgical site infections, but the anti-inflammatory mechanism is not fully understood (52). Subsequently, WANG et al. conducted further research and found that tranexamic acid prevents implant related infections by reducing biofilm formation in infected tissues (53). Several studies have revealed that TXA can repress inflammation by modulating cytokine levels to alter inflammatory markers in patients (54). Interestingly, in addition to its hemostatic properties, ORC creates a low pH environment to exert broad-spectrum antibacterial effects (55). A previous *in vitro* study highlighted that ORC had antibacterial activity against both Gram-positive and Gram-negative strains, including drug-resistant strains, and decreased the incidence of infectious events (56). In the current study, CRP and ESR in patients were monitored, which showed no statistically significant difference in inflammatory markers among the three groups. None of the patients developed early infections, and no statistical difference was noted in wound complications among the three groups. All of these results suggest that the intraoperative application of ORC is equally safe as compared to TXA. However, the experimental data did not detect relevant inflammatory factors, so the anti-inflammatory properties of these two materials were not explained.

Lower-extremity deep vein thrombosis is a common complication after TKA due to endothelial damage to the vessel wall and the hypercoagulable state of the blood, as well as the characteristics of many branches, small valves, and slow blood return of the venous vessels in the calf (57). In recent years, the perioperative management of TKA is gradually improving. In our study, no patients suffered from lower-extremity deep vein thrombosis. In terms of asymptomatic calf muscular vein thrombosis with higher incidence, no statistical difference was found among the three groups (*P* = 0.766). Notably, the incidence of postoperative lower-extremity swelling, dizziness, and vomiting was the highest in the TXA group. However, there was no statistical difference among the three groups. Maria et al. think that administration of high doses of tranexamic acid is associated with seizures and other adverse effects that increase the cost of care, and need to reevaluate and review whether the dosage, route and interval of administration, and methods used to control and analyze the anti-fibrinolytic mechanism of TXA are really optimal (9). Therefore, we believe that the use of tranexamic acid can cause drug reactions, and its dose, route, administration interval, and its suitable population need to be further studied. After 6 weeks of follow-up, no unplanned readmission occurred.

It is worth mentioning that, because of the ethical issues of protecting patients, an intravenous drip of TXA was used as a routine hemostatic regimen during surgery in this study which is used by many hospitals in actual treatment. Hence, this study evaluated the hemostatic effect of topical ORC and TXA in the context of actual therapeutic procedures for TKA, which may have more clinical value.

When it comes to cost, one previous study concluded that tranexamic acid is effective and cheap (58). However, compared to the costs incurred by our previous multiple applications of tranexamic acid (85.5 USD), there was no obvious increase in the cost of hospitalization after the use of ORC (88.3 USD), which did not cause any additional financial burden to the patient.

Tranexamic acid is also used in multiple modes in the clinic, such as systemic TXA and local application combined with epinephrine. Systemic tranexamic acid has become the choice of many surgeons in the last decades, with good results (9). However, whether the route and interval of administration is optimal is not yet conclusive and needs to be evaluated and reviewed using the latest research to improve the safety and efficacy of tranexamic acid in various surgical procedures. Recently, several literatures have reported the negative effects of local and intravenous administration of tranexamic acid on articular cartilage and ligament tissue (59, 60). Is the current use of tranexamic acid in large quantities and many times potentially dangerous? The local application of tranexamic acid combined with epinephrine has also been reported in several literatures (61, 62). The authors state that it reduces patient bleeding, lowers transfusion rates, and is a viable option, but long-term follow-up is needed to ensure its safety.

Of course, there are several limitations in the present study. First, in this study, the severity of knee osteoarthritis in the included patients was not graded, and different degrees of intraoperative osteophyte removal and soft tissue release might interfere with the amount of blood loss and affect the experimental results. Second, based on enhanced recovery after surgery guidelines, patients were hospitalized for a short time (3 days), and blood loss indicators were monitored only until postoperative day 3. Therefore, TBL might not peak. In addition, since the follow-up period was short, the effect of ORC on the long-term postoperative knee function of patients is unexclusive. Third, all of the cases in this study were recruited from a research center of Asia with a large surgical volume, limiting the widespread use of our conclusions in medical institutions in other areas of the world.

In conclusion, our prospective randomized controlled trial (RCT) highlights that, oxidized regenerated cellulose (ORC) can reduce postoperative invisible blood loss in total knee arthroplasty and achieve a hemostatic effect similar to topical tranexamic acid (TXA). This provides a safe and effective hemostatic option for patients with severe underlying diseases or contraindications to tranexamic acid.

## Data Availability

The original contributions presented in the study are included in the article/Supplementary Material, further inquiries can be directed to the corresponding authors.
